# Do school classrooms meet the visual requirements of children and recommended vision standards?

**DOI:** 10.1371/journal.pone.0174983

**Published:** 2017-04-03

**Authors:** Kalpa Negiloni, Krishna Kumar Ramani, Rachapalle Reddi Sudhir

**Affiliations:** 1 Medical Research Foundation, Sankara Nethralaya, Chennai, India; 2 Shanmugha Arts, Science, Technology & Research Academy (SASTRA) University, Thanjavur, India; 3 Elite School of Optometry, Chennai, India; Universita degli Studi di Firenze, ITALY

## Abstract

**Background:**

Visual demands of school children tend to vary with diverse classroom environments. The study aimed to evaluate the distance and near Visual Acuity (VA) demand in Indian school classrooms and their comparison with the recommended vision standards.

**Materials and methods:**

The distance and near VA demands were assessed in 33 classrooms (grades 4 to 12) of eight schools. The VA threshold demand relied on the smallest size of distance and near visual task material and viewing distance. The logMAR equivalents of minimum VA demand at specific seating positions (desk) and among different grades were evaluated. The near threshold was converted into actual near VA demand by including the acuity reserve. The existing dimensions of chalkboard and classroom, gross area in a classroom per student and class size in all the measured classrooms were compared to the government recommended standards.

**Results:**

In 33 classrooms assessed (35±10 students per room), the average distance and near logMAR VA threshold demand was 0.31±0.17 and 0.44±0.14 respectively. The mean distance VA demand (minimum) in front desk position was 0.56±0.18 logMAR. Increased distance threshold demand (logMAR range -0.06, 0.19) was noted in 7 classrooms (21%). The mean VA demand in grades 4 to 8 and grades 9 to 12 was 0.35±0.16 and 0.24±0.16 logMAR respectively and the difference was not statistically significant (p = 0.055). The distance from board to front desk was greater than the recommended standard of 2.2m in 27 classrooms (82%). The other measured parameters were noted to be different from the proposed standards in majority of the classrooms.

**Conclusion:**

The study suggests the inclusion of task demand assessment in school vision screening protocol to provide relevant guidance to school authorities. These findings can serve as evidence to accommodate children with mild to moderate visual impairment in the regular classrooms.

## Introduction

In India, children spend an average of seven to eight working hours per day at school for approximately 12 years [1]. The visual task in a classroom can be a sustained one involving near work, viewing chalkboard and listening to lecture or with a rapid change in fixation such as copying from the chalkboard or a material placed at near distance. These visual tasks can impose varying demands on the visual system of a child.

One of the initiations by the Government of India is to make elementary education universal across the country and provide inclusive education to children with disabilities [1]. Children with mild disabilities such as vision, hearing, communication, perceptual-motor, social-emotional, intelligence and adaptive behaviour are enrolled in a mainstream school and may require higher task demand. The process of imparting education in children becomes more involved as a student moves from elementary to higher grades, demanding additional facilities to the basic classroom unit. The Indian standard recommendation for school buildings provides standards for spatial and environmental needs of basic classroom [2]. However, there is limited evidence regarding the school infrastructure meeting the standards. The critical components of school infrastructure including lighting, ventilation, temperature, furniture, noise, colour and class size have been known to affect student’s comfort, learning, and performance [3–4].

Eye care professionals generally advise children with reduced vision to be seated in a front desk position for better visibility based on a high contrast visual acuity measure. There is a paucity of current information concerning the level of vision up to which a child with reduced vision can be accommodated in a mainstream school. Low refractive error correction in early schooling years is debatable with few practitioners considering it to be non-amblyogenic and others advising to prescribe considering the effect on academic performance [5–6]. The lack of uniformity in prescribing protocols highlights the need to understand the visual acuity demand placed on a child in classroom.

A literature search revealed only a limited number of studies evaluating the visual acuity demand of school classrooms and limited to primary school classrooms. The distance and near threshold visual acuity demand of primary school classrooms in the USA [7] (0.37 logMAR and 0.73 logMAR, grade 5) was similar to Australia [8] (0.33 logMAR and 0.72 logMAR, grade 5 and 6).

The aim of the current study was to evaluate the distance and near visual acuity demand in classrooms of different grade levels in Indian schools. The classroom parameters were compared to the government recommended standards.

## Materials and methods

The distance and near visual acuity demand in school classrooms was evaluated. This is a cross- sectional study conducted at schools located in Chennai city, South India during August 2014 to February 2015. We included all the schools which were a part of our regular school vision screening programme. The study was approved by Vision Research Foundation Review Board and Ethics Committee. Written informed consent was obtained from the school principal (Head of the school) prior to the study.

### Assessment of visual acuity demand

**Fig 1:**
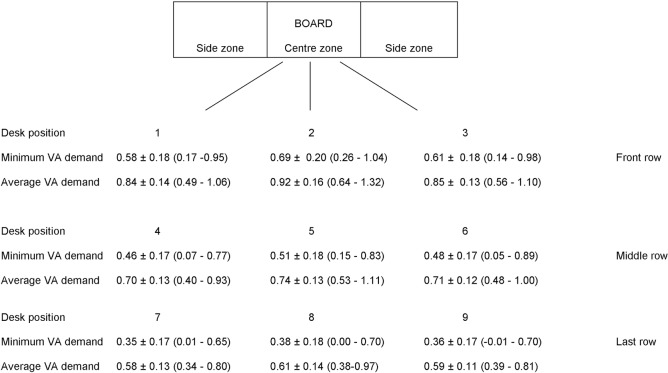
Categorization of desk positions and the minimum and average distance visual acuity demand (Mean±SD (range)) at different desk positions in 33 classrooms.

We randomly selected one class representing each grade in every school. Thirty three classrooms (grades eight to twelve) of eight schools were included in the study. The dimensions of chalkboard (height x width) and classroom (length x breadth) were measured. The gross area (in m^2^) occupied per student in classroom was evaluated by dividing the total area of classroom by the total number of students. The visual acuity demand was evaluated based on the size of the task (distance or near learning material) and viewing distance. The teacher’s writing on the chalk board and presentations (videos) on the smart board were generally used for distance tasks, while textbook print and student’s handwriting on notebooks were primarily used for near tasks. For distance visual acuity demand, the vertical height of teacher’s handwriting on the chalkboard in each classroom was measured using a millimetre scale and care was taken to avoid parallax errors. Smart boards were available in 6 measured classrooms (18%) and were used occasionally for projecting videos. Due to variability in handwriting (consistency) on a chalkboard, a minimum of 30 letters (centre and side positions) was measured in each classroom. The height of the lowercase letter “x” was measured excluding the ascenders and descenders. Capital letters were excluded and small case letters in “English” language were included. All the measurements were taken by a single observer. The height, width and positioning of chalkboard in each classroom was recorded. School classrooms had varying dimensions and varying number of desks (rows and columns). Based on the total number of desks in a classroom, desk positions were categorised as front, middle and last row with the middle desk position chosen based on the total number of rows in the classroom. The extreme (left and right column) side desks and the desks in the centre of classroom (in front of the board) were chosen. The categorisation of desk positions (1 to 9) is shown in Fig 1. The distance from the chalkboard (centre and side) to each of the desk position 1 to 9 was measured.

The visual acuity demand in Snellen equivalent and logMAR was calculated with the vertical height of letter and its viewing distance. The critical detail was taken as one fifth of the letter height and the method adopted to calculate the visual acuity demand was the same as previously described and used by Hug et al [7] and Narayanasamy et al [8]. The maximum distance visual acuity threshold demand was calculated based on the smallest letter size and longest viewing distance in each classroom. At each desk position, the minimum and average visual acuity demand across the measured classrooms was evaluated based on the smallest and average letter size respectively.

The near visual acuity demand was evaluated by measuring the smallest vertical lower case letter height of learning material and its viewing distance. The letter height of text book print in different grades and a random sample of student’s handwriting in their notebooks were measured using a millimetre scale. The near working distance of 80 randomly selected children from each of the grade 5 to 12 (ten students from each grade) was evaluated while children were performing near work in their classroom by a single observer. The near visual acuity threshold demand was calculated adopting the same method used for distance acuity demand. In children, to perform a sustained near task (fluent reading), an acuity reserve of 2.5 times the threshold visual acuity is suggested [9]. The actual near visual acuity demand was calculated including the acuity reserve [8].

### Statistical analysis

All the data was entered in Excel (Microsoft Office 2013) and the statistical analysis was performed using IBM Statistical Package for Social Sciences (SPSS) version 20. Descriptive analysis of classroom dimensions, viewing distance, size of visual task, and the distance and near visual acuity demand was performed and the values are presented as mean±standard deviation (minimum, maximum). Independent t test and one way ANOVA was performed to compare between different grades (categorised as Grades 4 to 8 and Grades 9 to 12) and different schools (n = 8) respectively and p-value less than 0.05 was considered as statistically significant.

## Results

A total of 33 classrooms (35±10 students per room) in eight schools were included in the study. The mean dimension (height x width) of the chalkboard was 1.3±0.18 m (1, 1.7) x 2.8±0.99 m (1.2, 6.1). The average length x breadth of the classrooms was 6.1±0.83 m (4.2, 8) x 5.5±1.1 m (2.8, 6.8) and was rectangular in shape. The number of rows and columns (desk positions) in each of the measured classrooms varied with a range of 3 to 7 and 2 to 4 respectively. The measured classrooms were occupied by an average of 35±10 students (20, 58) and one teacher. The mean gross area of classroom per student in the 33 measured classrooms was 1.01±0.36 m^2^ (0.47, 1.73) and 70% (n = 12) classrooms had gross area lesser than 1.26 m^2^ (Bureau of Indian Standards recommendation) [2]. The parameters in measured classrooms and the recommended standards by Indian government are compared in Table 1.

**Table 1:** Comparison of variables in measured 33 classrooms to the Indian Government standard recommendations.

Variable	Standard recommendations by the Government of India [2]	Mean±SD of existing levels in measured classrooms	Percentage of classrooms with levels beyond recommendations
Number of students per classroom	40	35±10	24 (>40 students)
Chalkboard dimension (length x width in metre)	1.2 x 2.4	1.3±0.18 x 2.8±0.99	21 (less than 1.2 x 2.4)
Distance from the board to front desk position in metre	2.2	2.76±0.66	82 (>2.2m)
Gross area of classroom per student in m^2^	1.26	1.01±0.36	70 (<1.26 m^2^)

### Visual acuity demand

**Fig 2:**
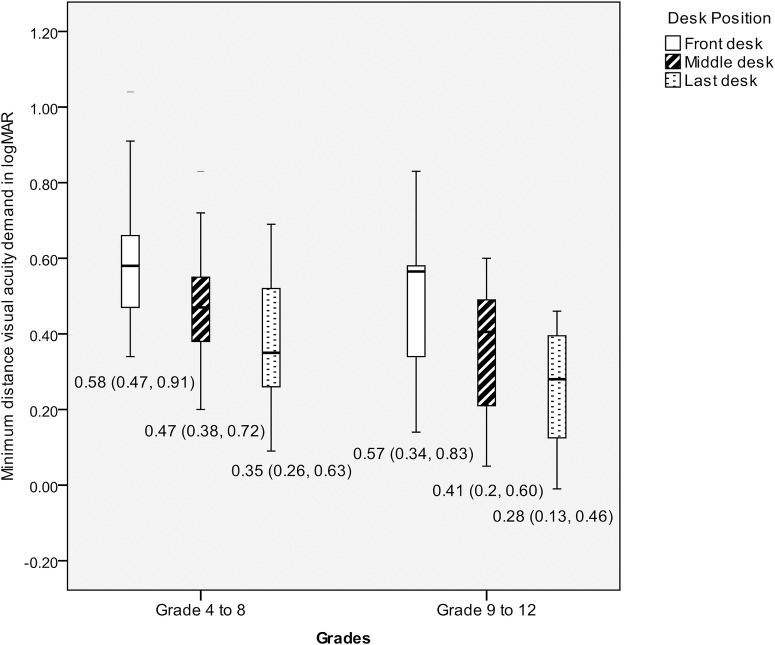
Box plot representing the comparison of minimum distance visual acuity demand at different desk positions (front, middle and last row) between grades 4 to 8 and grades 9 to 12.

The vertical height of the teachers’ handwriting, with a range of 20 to 32 letters per chalkboard zone was measured. The mean vertical height of all the letters was 3.43 cms (95% CI, 1.51–5.35 cms). The smallest letter size (n = 33 classrooms) on chalkboard was 2.0 ±0.56 cms, with no significant difference between grade groups (grades 4 to 8 and grades 9 to 12) and between 8 recruited schools (Table 2). The average distance from chalkboard to front row (desk position 2) and the longest viewing distance in classroom are described in Table 2. The average visual acuity demand of children was 0.74 logMAR (95% CI, 0.47–1.00 logMAR). Based on the smallest letter size and longest viewing distance in a classroom, the average maximum distance visual acuity threshold demand was 0.31±0.17 logMAR. An increased threshold demand range (-0.06, 0.19 logMAR) was noted in 7 classrooms (21%). The average maximum distance VA threshold demand in grades 4 to 8 and grades 9 to 12 was 0.35±16 logMAR (0.07, 0.62) and 0.24±0.16 logMAR (-0.06, 0.45) respectively. There was no significant difference in the maximum distance threshold demand and front desk visual acuity demand between grades and different schools (Table 2). The mean (range) of the minimum and average visual acuity demand at different desk positions (1 to 9) is shown in Fig 1. There was no statistically significant difference in VA demand between grades 4 to 8 and grades 9 to 12 in the first row (p = 0.132) and the middle row (p = 0.053). There was statistically significant difference noted in VA demand between grades 4 to 8 and grades 9 to 12 in the last row (p = 0.040). The VA demand at different desk positions between grades 4 to 8 and grades 9 to 12 in shown in Fig 2.

**Table 2:** Average letter size, viewing distance and visual acuity demand, and comparison between different grades (grade 4 to 8 and grade 9 to 12 group) and schools (n = 8) in 33 classrooms.

Variable	Mean (SD)	Range	Between grade groups (Independent t test statistic, p value)	Between schools (One way ANOVA, F (7,25) statistic, p value)
Smallest letter height (cms)	2.0 ±0.56	1–3.10	0.136	0.392
Distance from centre of board to front row (m)	2.76±0.66	1.8–4.14	0.244	0.139
Longest distance student seated (m)	6.56±1.16	4.1–8.7	0.128	0.076
Maximum distance VA threshold demand (logMAR)	0.31±0.17	-0.06, 0.62	0.055	0.113
Minimum Visual demand at front row (desk position 2) in logMAR	0.56±0.18	0.14–1.04	0.132	0.132

The mean near working distance of children (grades 5 to 12) was 25±2.86 cms (20, 35) and there was no statistically significant difference in working distance between grades 5 to 8 (24.9±2.91) and grades 9 to 12 (25.1±2.84), p = 0.699. Based on lower case size of near tasks in classroom and habitual working distance of children taken as 25cms, the average near acuity threshold demand was 0.83±0.14 logMAR (0.44, 1.04). The average actual near acuity demand calculated considering the acuity reserve was 0.44±0.14 logMAR (0.04, 0.64). The average near acuity threshold demand in grades 5 to 8 and grades 9 to 12 was 0.82±0.16 logMAR (0.44, 1.04) and 0.85±0.12 logMAR (0.62, 1.04) respectively. The average actual near acuity demand considering the acuity reserve in grades 5 to 8 and grades 9 to 12 was 0.42±0.16 logMAR (0.04, 0.64) and 0.45±0.12 logMAR (0.22, 0.64) respectively.

The smallest lower case letter height in school text books was 2mm in grades 6 to 12 and the equivalent visual acuity demand calculated was 0.74 logMAR at 25cms viewing distance (Actual demand considering acuity reserve, 0.34 logMAR).

## Discussion

This is the first study to evaluate the distance and near visual acuity demand in different grades of classrooms in Indian schools. The distance and near visual task demand in school classrooms of Chennai city was 0.31 logMAR and 0.44 logMAR respectively. Previous studies have evaluated the visual demand in primary school classrooms. An increased visual acuity demand was reported with increasing grade level in primary classrooms by a US based study reporting an average distance visual acuity demand of 0.70 to 1.18 logMAR for grades KG to 2 and 0.48 to 0.70 for grade 3 to 5 [7]. A similar study conducted in Australian school classrooms reported the maximum distance VA threshold demand of 0.33logMAR (range, 0.06 to 0.64) in grade 5 and 6 students [8]. The maximum distance VA threshold demand (0.31 logMAR) in the measured Indian classrooms was similar to previous studies, highlighting a higher visual task demand in school classrooms. Our study reports a distance visual acuity threshold demand of 0.35 logMAR in grades 4 to 8 and 0.24 logMAR in grades 9 to 12.

With the advancement in smart board systems, most of the teachers in Indian school still primarily prefer a chalkboard (black/green) system. Other factors such as width of chalk letters, contrast of letters, legibility and light levels can influence the visibility of chalkboard writing in addition to the target size and can further increase the distance visual acuity demand in a classroom. Increased light levels on the board can cause glare and reduce visibility. Resolution acuity measure includes the stroke width of a letter (critical detail) to determine the visual acuity. The critical detail on a letter was arbitrarily taken as one fifth of the letter height. The limitation of the current study is representing the acuity estimate as visual demand and not the actual resolution acuity to recognize the letters. In India (Tamil Nadu), the mode of education system adopted for elementary school (grades 1 to 4) includes activity based learning system which has a child centered approach by the use of self learning materials such as study cards [1]. These learning materials can be customized by increasing the font in a good contrast material, based on the visual needs of children, especially with visual impairment.

Kumaran et al (2015) studied the vision- related quality of life in school children (South India) with uncorrected refractive error (URE) [10]. Based on focused group discussion, complaints and symptoms reported by children with URE were difficulty in distant vision to view blackboard, headache, eye strain, eye pain, watering of eyes, difficulty in recognizing faces especially in the dark, difficulty in participating in sport activities and hesitance to participate in co-curricular activities and competitions. Most of these symptoms were noted to be improved, in addition to better academic performance following first time refractive correction. Clinical opinion varies in prescribing for low refractive errors in children. Experts have provided guidelines for prescribing refractive error in children based on their clinical opinion and research evidence. The minimum cut off for refractive error correction suggested for school children without symptoms are 1.50D for hyperopia, 0.75D for astigmatism 0.50D for myopia [5, 11]. Visual acuity of 0.1 log unit (one line) corresponds to 0.25D of spherical refractive error. This relationship can be varied due to factors such as pupil size, illumination, target type and instruction to the patient [12]. With an increased visual task demand of 20/40 based on the current study, a child with uncorrected low refractive error would have increased task demand and need correction. School vision screening has a cut off visual acuity of 0.20 logMAR (20/30) [13]. In the current study, 21% of classrooms had distance visual acuity threshold with a demand greater than 0.20 logMAR (range -0.06, 0.19 logMAR) indicating that children who pass the screening test based on this criteria (0.20 logMAR) are at risk of visual stress in these classrooms.

Children spend more than half of the school day performing sustained near tasks. To be able to perform near tasks as long as 30 to 40 minutes (average lecture hour) [1], a child needs to sustain an accommodative level of 2.50 D or more at 40cms. The working distance of children was found to be in closer range (25cms), indicating an accommodative demand of 4D and increased convergence demand. The physiological demand placed on the visual system while performing near task includes the flexibility and amplitude of accommodative-convergence system and visual-sensory motor integration [14]. The physiological demands can be affected by various environmental factors such as poor lighting, poor contrast materials and reduced size of the task. If the visual system fatigues, the psychological system will also fatigue affecting attention and learning [14, 15]. Children with accommodative or vergence anomalies, need appropriate management in addition to modifying the ergonomic factors in a classroom to avoid visual stress. Shankar et al (2007) have reported uncorrected hyperopia (>2.00DS) in children aged 4 to 7 years with reduced performance in letter and word recognition, receptive vocabulary and emergent orthography [16]. In simulated refractive error studies, children with low levels of bilateral hyperopia (+2.50DS) and bilateral astigmatism (1.50 DC) doing sustained near work (20 minutes) had reduced performance in academic-related measures (reading, visual information processing and reading related eye movement tests) [17, 18]. Early identification and correction of refractive error can avoid its effect on learning and academic performance. Few studies suggest longer hours of patching for children with amblyopia and this can necessitate patching to be performed during schools hours [19, 20]. The level of visual demand placed in a classroom needs to be known to advise patching during school hours.

The average school day in Indian schools is 7 to 8 hours with children performing various visual tasks such as copying from the board, reading, writing and using computer. The Government of India has initiated Sarva Shiksha Abhiyan (SSA) to make elementary education universal across the country and provide an inclusive education to children with disabilities [1]. SSA recommends a barrier free physical environment for children with visual impairment which includes reducing the distance from the chalk board to first row of students desk (< 2.2m), chalkboard dimension of 1.2x2.4m, student ratio of 1:40 and gross area of classroom per student [1,2].

Children with any type of sensory impairment or academic difficulty are customarily seated in the front row. Our findings indicate that children with mild to moderate visual impairment can be seated in the front row to perform visual task with ease in their classroom. With the standard distance from the board to front desk recommended as lesser than 2.2m, the font of teacher’s writing should be more than 3.2 cms (lower case size) to meet the visual demand of 1.0 logMAR. We found an average distance of 2.76 m from the centre of board to first row of student’s desk and minimum size of the font as 1 cm, highlighting the need for larger font to be written on the board or reducing the viewing distance to relieve visual stress in children. Eye care professionals should also consider the classroom environment demand placed on a child before advising a low vision aid, amblyopia therapy during school hour or deciding prescribing children with low refractive errors. In a school eye screening, in addition to the refractive error correction, recommendations regarding the font size and distance to be seated from the board can be provided to aid children with visual impairment. Simple stencil markings on the corners of board can aid teachers maintain the required font size while writing on the board.

## Conclusion

School vision screening protocol can include the assessment of task demand and provide appropriate recommendations to the school authorities. These findings provide evidence based recommendations to accommodate children with mild to moderate visual impairment in the regular classrooms.

## Supporting information

S1 Data (XLSX)
